# Supplementary figures and images for: Unveiling dynamic hepatocyte plasticity in HepaRG cells with a dual CYP reporter system
Source: PLoS One. 2024 Nov 11;19(11):e0308694. doi: 10.1371/journal.pone.0308694 (PMC11554142; doi:10.1371/journal.pone.0308694)

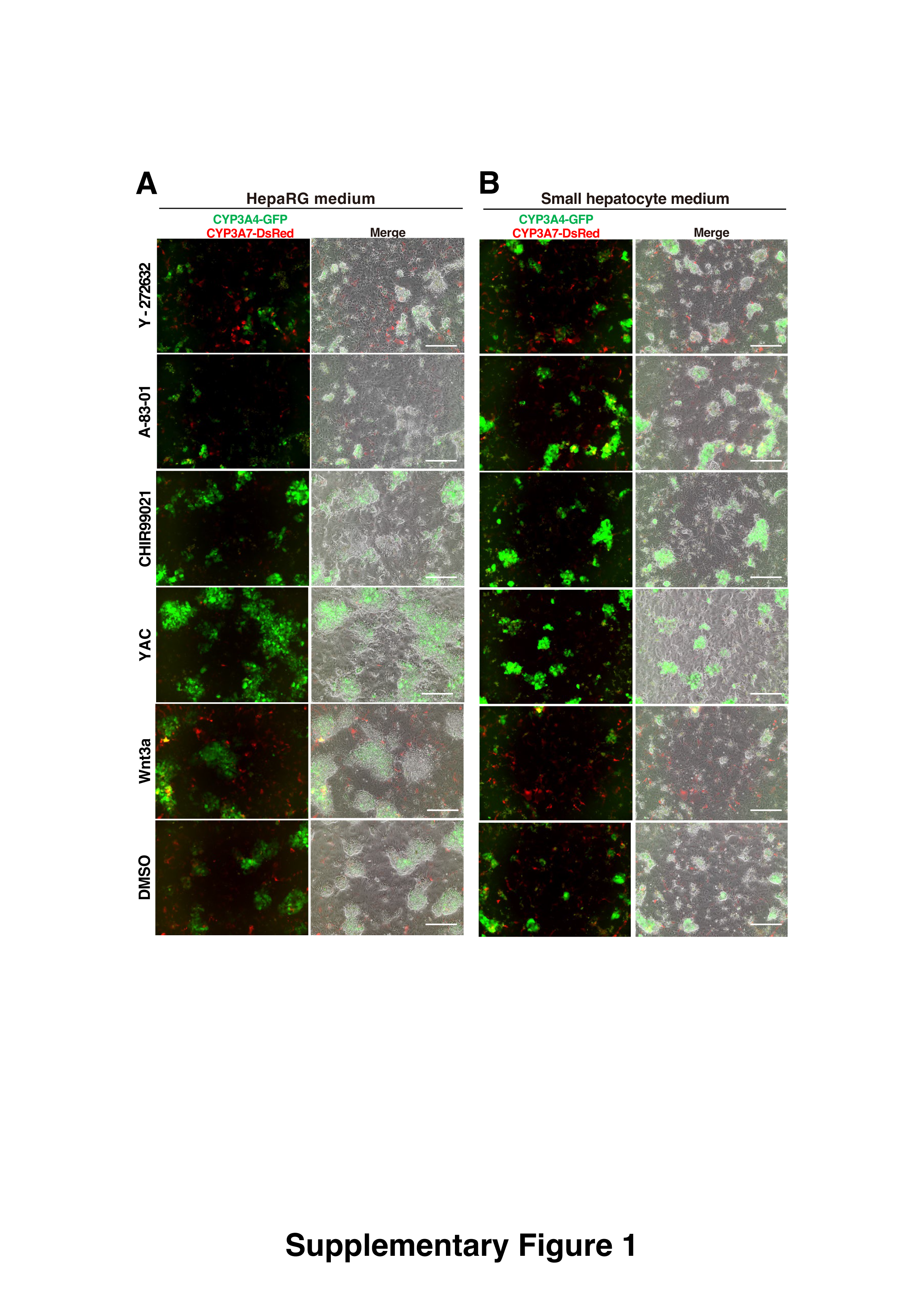

Supplement: S1 Fig — (A,B) Representative images of de-differentiated cells with chemicals in HepaRG medium (A), and in Small hepatocyte medium (B). (TIF) [file pone.0308694.s001.tif]

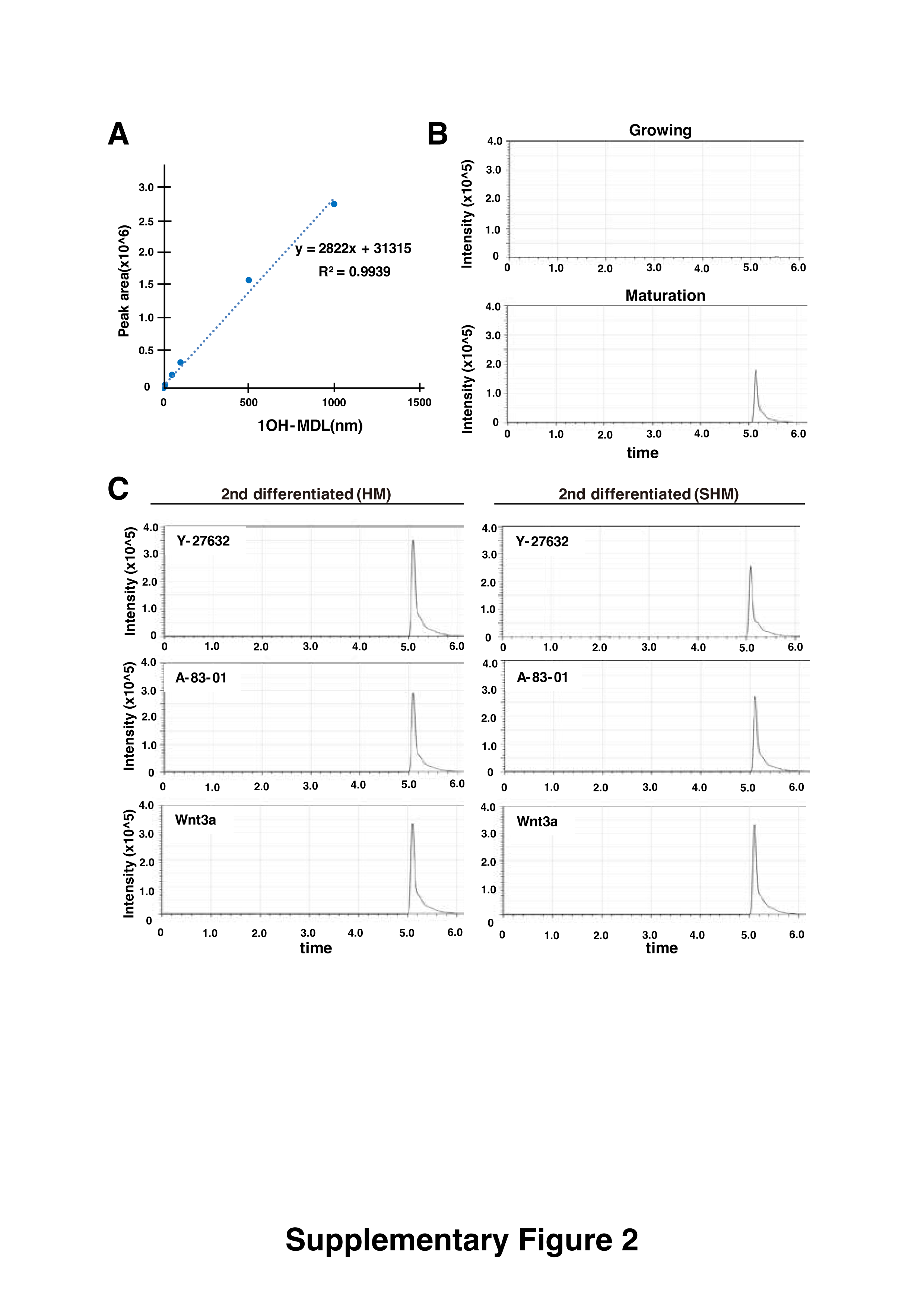

Supplement: S2 Fig — (A) A calibration curve for 1’-OH Midazolam using LC-MS/MS. (B) The LC-MS/MS peak of 1’-OH midazolam used to measure the CYP3A4 enzyme activity in HepaRG cells. On days 7 of proliferation and day 2 of maturation, midazolam, an enzyme target of CYP3A4, was administered to cells. (C) The LC-MS/MS peak of second round differentiated cells. (TIF) [file pone.0308694.s002.tif]
