# Supplementary material for: Unveiling dynamic hepatocyte plasticity in HepaRG cells with a dual CYP reporter system
Source: PLoS One. 2024 Nov 11;19(11):e0308694. doi: 10.1371/journal.pone.0308694 (PMC11554142; doi:10.1371/journal.pone.0308694)
Supplement: Attachment — Submitted filename: Rebuttal letter.docx [file pone.0308694.s005.docx]

**Rebuttal Letter**

Dear Editor,

Thank you for the opportunity to revise our manuscript titled "**Unveiling Dynamic Hepatocyte Plasticity in HepaRG Cells with a Dual CYP Reporter System.**" We appreciate the valuable feedback provided by the editor and reviewer and have carefully addressed all comments. Below is a summary of the major revisions made to the manuscript:

**Reviewer #1:**

Comment 1: "I have only one suggestion to the authors: to elaborate on the differences between the HepRG cell line, primary hepatocytes in culture, and hepatocytes in vivo and on how their results can be used to improve drug testing."

Response: We appreciate this valuable suggestion. In the revised manuscript, we have expanded the introduction section to compare the characteristics of HepaRG cells with primary hepatocytes in culture and hepatocytes in vivo (lines 69-81).

Comment 2: "Figures 2 and 3 contain a common misprint in the phrase 'small hepatcyte medium.'"

Response: We apologize for this oversight. The typo has been corrected to “small hepatocyte medium” in both Figures 2 and 3.

**Editorial Requests:**

1. **Funding Statement Update**: We have removed the funding information from the Acknowledgments section.
2. **Data Availability Statement**: We will make the full dataset available upon acceptance. The release date has been updated to Oct 1, 2024. We confirm that the data will be shared in compliance with PLOS ONE’s data-sharing policy.
3. **Manuscript Formatting**: We have ensured that the revised manuscript adheres to PLOS ONE’s formatting guidelines. The appropriate file naming conventions have been used, and changes are tracked in the highlighted version of the manuscript.

We are confident that these revisions address all the concerns raised, and we believe the revised manuscript now meets the criteria for publication in PLOS ONE. We thank you again for your time and consideration.

Sincerely,

Shinpei Yamaguchi
